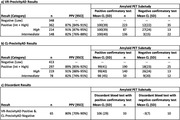# Use of a blood‐based amyloid test for screening in INVOKE‐2: a phase 2 randomized, double‐blind, placebo‐controlled study evaluating AL002 in early Alzheimer’s disease

**DOI:** 10.1002/alz.095646

**Published:** 2025-01-09

**Authors:** Brady Burgess, Tuan Nguyen, Adam Simmons, Ruchi Gupta, Tiffany Chow, Gary Romano

**Affiliations:** ^1^ Alector, South San Francisco, CA USA

## Abstract

**Background:**

The PrecivityAD® blood‐test (C2N Diagnostics) predicts cerebral amyloidosis due to Alzheimer’s Disease (AD). PrecivityAD® incorporates plasma Aβ42/40, age and apoE proteotype into an algorithm that generates an amyloid probability score (APS) corresponding to the likelihood of a positive amyloid PET scan, defined as ≥25 centiloid (CL). A prototype PrecivityAD algorithm validated against amyloid PET status scored by visual read (VR) was used to improve screening efficiency in INVOKE‐2, a phase 2 randomized, double‐blind, placebo‐controlled trial evaluating TREM2‐activating antibody AL002 in early AD.

**Methods:**

VR‐validated PrecivityAD (VR‐PrecivityAD) used refined thresholds defined as low (VR‐APS = 0‐31), intermediate (32‐57) or high (58‐100) likelihood of amyloid positivity. CL‐validated PrecivityAD (CL‐PrecivityAD) results were scored using established thresholds for low (CL‐APS = 0‐35), intermediate (36‐57) or high (58‐100) likelihood of amyloid positivity. INVOKE‐2 screening subjects meeting inclusion criteria for Clinical Dementia Rating Global Score (CDR‐GS), Mini‐Mental State Examination (MMSE), and Repeatable Battery for the Assessment of Neuropsychological Status Delayed Memory Index (RBANS‐DMI) (CDR‐GS = 0.5‐1, MMSE≥20, RBANS‐DMI≥95) were required to have a positive VR‐PrecivityAD blood‐test (intermediate or high VR‐APS result) prior to a confirmatory amyloid PET VR or CSF test to establish eligibility. Positive predictive value (PPV) was defined as number of individuals with confirmed positive blood‐tests as a percentage of all positive blood‐tests.

**Results:**

Positive VR‐PrecivityAD results were reported for 51% of individuals (362/710) with a PPV of 87% overall, 91% for high or 82% for intermediate results (Table). Comparison of VR‐APS against CL‐APS results indicated the INVOKE‐2 positivity threshold (VR‐APS≥32) was equivalent to an effective CL‐APS threshold of ∼25, lower than the established CL‐PrecivityAD threshold (CL‐APS≥36). In this comparison 58% of individuals would have scored negative on CL‐PrecivityAD compared to 49% observed with VR‐PrecivityAD. Importantly, 80% (52/65) of discordant individuals with positive VR‐PrecvityAD and negative CL‐PrecivityAD results had a subsequent positive confirmatory test (Table).

**Conclusions:**

The prototype VR‐PrecivityAD blood‐test was highly predictive of amyloid positivity, confirmed by VR‐PET or CSF, among INVOKE‐2 screening subjects. Use of a lower VR‐APS threshold was associated with fewer false negatives without a meaningful increase in false positives when compared to the standard CL‐PrecivityAD threshold in the INVOKE‐2 screening population.